# Editor's Message

**DOI:** 10.29173/jchla29603

**Published:** 2021-12-01

**Authors:** Alanna Campbell

*[Français en suite]*

Hello and welcome to our December 2021 issue of JCHLA/JABSC! As we continue to persevere through this pandemic I want to recognize all of you, our readers and authors, who continue to support health sciences library users – many of whom are current or future frontline workers.

I also want to take a moment to thank Sandra McKeown our former Editor-in-Chief for all her efforts on the editorial board the last three years. Sandra led the editorial board in developing JCHLA/JABSC editorial policies and practices to address systemic racism and improve equity, diversity, and inclusion. As mandated, at our first meeting of the year held in September, we met to discuss anti-racism and equity, diversity and inclusion (EDI) professional development for all members of the editorial team. At this meeting we shared educational opportunities we had undertaken and resources that we encourage you, our readership, to consider integrating into your own professional development practice.

Courses and workshops included, Library Juice: Cultural Competence for Librarians and CHLA: Exploring your Identity with Cultural Humility. Online resources such as Killing Me Softly, a game about microaggressions and the Antiracism Toolkit for Allies were discussed. Kimberlé Crenshaw’s books, such as *On intersectionality: The essential writings*, were also recommended at this meeting. If you have recommendations for our team please send them to editor@chla-absc.ca. We also want to continue to encourage submissions on topics of anti-racism, racial injustice, and equity, diversity and inclusion pertaining to health sciences librarianship.

In this issue, we have three research articles. Phinney and Kiester address if Canadian librarian’s are supporting medical student health and wellness. Babb reports on Canadian academic’s use of predatory journals. While McKewon, Mir, Ritonja and Soleas look at systematic review support in Ontario medical schools. Also included are two book reviews, *A history of medical libraries and medical librarianship: From John Shaw Billings to the digital era* and *Planning and promoting events in health sciences libraries: Success stories and best practices*. We finish off with two product reviews assessing PICO Portal and H5P.

I hope you enjoy the latest issue of JCHLA/JABSC!

**Alanna Campbell**

*JCHLA/JABSC Editor-in-Chief*

*Email: editor@chla-absc.ca*

Bonjour et bienvenue à notre numéro de décembre 2021 de JABSC/JCHLA ! Alors que nous continuons à persévérer dans cette pandémie, je tiens à vous rendre hommage à vous tous, nos lecteur.rices et auteur.es, qui continuez à soutenir les utilisateur.rices des bibliothèques en sciences de la santé - dont beaucoup sont des travailleurs de première ligne actuels ou futurs.

Je souhaite également prendre un moment pour remercier Sandra McKeown, notre ancienne rédactrice en chef, pour tous les efforts qu'elle a déployés au sein du comité de rédaction au cours des trois dernières années. Sandra a dirigé ce comité pour l'élaboration de politiques et de pratiques éditoriales du JABSC/ JCHLA afin de lutter contre le racisme systémique et améliorer l'équité, la diversité et l'inclusion. Comme prévu, lors de notre première réunion de l'année en septembre nous nous sommes réunis pour discuter de l'antiracisme et du développement professionnel en matière d'équité, de diversité et d'inclusion (ÉDI) pour tous les membres de l'équipe éditoriale. Lors de cette réunion, nous avons partagé les possibilités de formation entreprises et les ressources que nous vous encourageons à envisager d'intégrer à votre propre pratique de développement professionnel.

Les cours et ateliers comprenaient, entre autres, Library Juice: Cultural Competence for Librarians et *CHLA: Exploring your Identity with Cultural Humility*. Des ressources en ligne telles que *Killing Me Softly*, un jeu sur les microagressions, et le *Antiracism Toolkit for Allies* ont été discutées. Les livres de Kimberlé Crenshaw, tels que *On intersectionality : The essential writings*, ont aussi été recommandés lors de cette réunion. Si vous avez des recommandations pour notre équipe, veuillez les envoyer à mailto:editor@chla-absc.ca. Nous souhaitons également continuer à encourager les soumissions d’articles sur les thèmes de l'antiracisme, de l'injustice raciale, de l'équité, de la diversité et de l'inclusion en rapport avec la bibliothéconomie en sciences de la santé.

Dans ce numéro, nous avons trois articles de recherche. Phinney et Kiester se demandent si les bibliothécaires canadiens soutiennent la santé et le bien-être des étudiants en médecine. Babb fait état de l'utilisation de revues prédatrices par les universitaires canadien.nes. McKewon, Mir, Ritonja et Soleas se penchent sur le soutien aux revues systématiques dans les facultés de médecine de l'Ontario. Deux comptes rendus de livres sont également inclus , *A history of medical libraries et medical librarianship: From John Shaw Billings to the digital era* et *Planning and promoting events in health sciences libraries: Success stories and best practices*. Nous terminons avec deux évaluations de produits qui testent le PICO Portal et H5P.

J'espère que vous apprécierez le dernier numéro de JABSC/JCHLA!

**Alanna Campbell**

*JCHLA/JABSC Rédactrice en chef*

*Courriel: editor@chla-absc.ca*

